# Transient expression vectors for functional genomics, quantification of promoter activity and RNA silencing in plants

**DOI:** 10.1186/1746-4811-1-13

**Published:** 2005-12-18

**Authors:** Roger P Hellens, Andrew C Allan, Ellen N Friel, Karen Bolitho, Karryn Grafton, Matthew D Templeton, Sakuntala Karunairetnam, Andrew P Gleave, William A Laing

**Affiliations:** 1 HortResearch, Mt Albert Research Centre, Private Bag 92169, Auckland, New Zealand

## Abstract

**Background:**

We describe novel plasmid vectors for transient gene expression using *Agrobacterium*, infiltrated into *Nicotiana benthamiana *leaves. We have generated a series of pGreenII cloning vectors that are ideally suited to transient gene expression, by removing elements of conventional binary vectors necessary for stable transformation such as transformation selection genes.

**Results:**

We give an example of expression of heme-thiolate P450 to demonstrate effectiveness of this system. We have also designed vectors that take advantage of a dual luciferase assay system to analyse promoter sequences or post-transcriptional regulation of gene expression. We have demonstrated their utility by co-expression of putative transcription factors and the promoter sequence of potential target genes and show how orthologous promoter sequences respond to these genes. Finally, we have constructed a vector that has allowed us to investigate design features of hairpin constructs related to their ability to initiate RNA silencing, and have used these tools to study *cis*-regulatory effect of intron-containing gene constructs.

**Conclusion:**

In developing a series of vectors ideally suited to transient expression analysis we have provided a resource that further advances the application of this technology. These minimal vectors are ideally suited to conventional cloning methods and we have used them to demonstrate their flexibility to investigate enzyme activity, transcription regulation and post-transcriptional regulatory processes in transient assays.

## Background

*Agrobacterium tumefaciens *is the primary tool used to generate transgenic plants [1]. During early stages of co-cultivation, single-stranded T-DNA is transferred from the bacteria to plant cells [2]. Once moved into the plant cell by bacterial and plant encoded proteins [3,4], this T-DNA becomes double-stranded and migrates to the nucleus. Only a small percentage is integrated into the host chromosomes leading to stably transformed cells that can subsequently be regenerated into transgenic plants. Although the long-term fate of the T-DNAs that do not integrate into the chromosomes is unclear, for a time, these pieces of DNA are transcriptionally competent; this is the basis of the *Agrobacterium*-mediated transient expression systems [5]. While *Agrobacterium*-mediated stable plant gene transformation requires binary vectors that allow plasmid manipulation in both *E. coli *and *Agrobacterium *and a selectable marker to recover transformed plants [1], no selectable marker is needed for transient expression. Omission of the selectable marker allows the cloning vectors to be smaller and easier to handle (e.g. less chance of duplicate restriction sites occurring) and may lead to increased frequency of plasmid ligation and bacterial transformation [6].

We have constructed a series of binary cloning vectors that have been specifically designed for transient gene expression in plant cells. Using plants as an expression system offers several advantages over prokaryotic or non-plant expression systems. For instance, genes that contain introns are processed and both subcellular targeting and post-translational modifications are possible. In addition, components necessary for transcriptional initiation, RNA processing, and translation initiation are already present in the plant.

In this study, we describe our plasmid vectors and transient gene expression system, drawing on examples of (i) assigning function to a heme thiolate (TH)-P450 gene, (ii) identifying a transcription factor target promoter, and (iii) exploring the role of RNA processing in dsRNA hairpin-induced RNA silencing.

Proteins of the HT-P450 class of genes are of particular importance to secondary metabolism. They catalyse a NADP-dependent hydroxylation step on a variety of plant metabolites that allows for modification of the base compound (e.g. terpene, phenyl propanoid) by other enzymes such as methyl transferases or alcohol acyl transferases. Heme thiolate-P450s are one of the largest families of enzymes in plants; there are 246 HT-P450 genes in the *Arabidopsis *genome: Arabidopsis Cytochrome P450))[7], though very few have been functionally characterised [8]. As these enzymes are membrane bound and require an NADPH HT-P450 reductase (EC 1.6.2.4) for activity, assaying these enzymes *in vitro *is difficult. Although yeast expression systems have been developed that allow these genes to be analysed [9], we show that our transient expression system can be used to assay the apple homologue of the HT-P450, cinnamic acid 4-hydroxylase, MdC4H1 (EC 1.14.13.11). Cinnamic acid is a metabolite in the phenyl propanoid pathway, a key pathway in plants leading to, among others, the production of lignin, lignan, flavonoids and anthocyanins [10].

Transcription factors (TF) are a large class of genes with DNA binding motifs [11]. Mechanistically these proteins bind to sequence elements within a gene's promoter and regulate transcription. TFs are able to coordinately regulate complex developmental processes or control entire metabolic pathways [11]. There are over 1400 known TFs in the *Arabidopsis *genome [11] and identifying the targets for each of these is a challenging task. We have taken advantage of a simple, commercial dual luciferase assay system that allows expression of both the target promoter controlling expression of the firefly luciferase (LUC) reporter gene and a control promoter (CaMV 35S) regulating expression of the *Renilla *luciferase (REN) reporter gene. We were able to determine the relative effectiveness of different TFs in stimulating expression of a reporter promoter-gene sequence construct.

Both protein over-expression and genetic approaches have benefited from recent advances in RNA silencing, a sequence-specific RNA degradation mechanism [12]. There are, however, aspects of RNA biochemistry, such as the effect of RNA processing, which may influence gene silencing, that remain poorly understood [13]. There are several RNA processing events such as intron-mediated enhancement [14], that are known to act post-transcriptionally, and a link between RNA processing and gene silencing remains possible. Using the transient assay system with dual reporter genes we were able to investigate hairpin expression constructs that differ in the intron configuration and speculate on the role RNA processing may play in influencing the expression of neighboring genes.

## Results and Discussion

### Vector constructs for over expression

**Figure 1:**
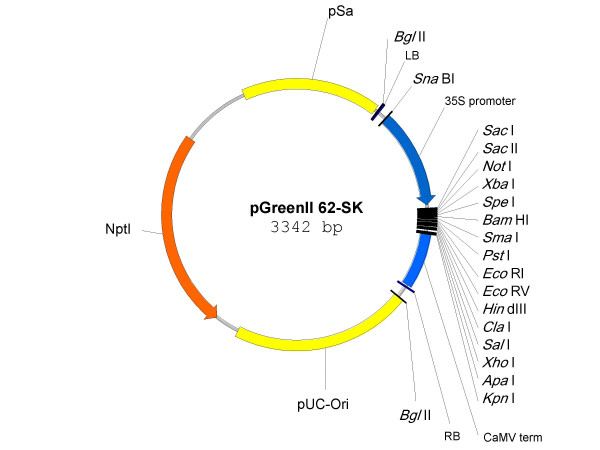
Plasmid map of the transient expression vector pGreenII 62-SK.

**Figure 2:**
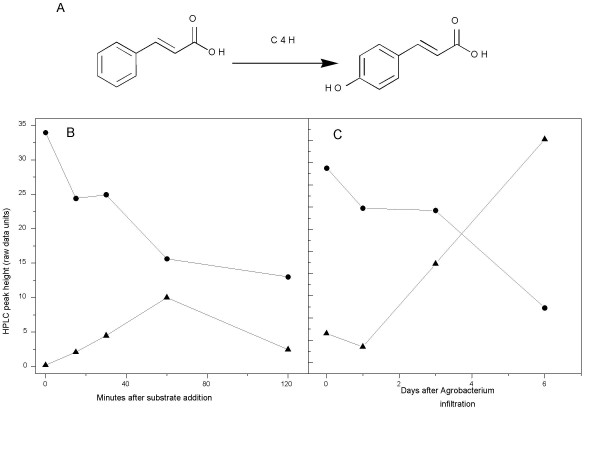
A, conversion of cinnamic acid to p-coumaric acid by the action of a heme thiolate P450; AdC4H. B, HPLC peak height of (▲) coumaric acid and (●) p-cinnamic acid at time points (minutes) after the infiltration of coumaric acid substrate. C, HPLC peak height of (▲) coumaric acid and (●) p-cinnamic acid at time points (days) after infiltration of Agrobacterium into plant leaves.

In the second step of the phenyl propanoid pathway, cinnamic acid is hydroxylated at the four position (see Fig. 2A) by HT-P450 hydroxylase, C4H (EC1.14.13.11), to produce p-coumaric acid. An apple orthologue, MdC4H1, was identified from the HortResearch apple EST database by sequence similarity [15] to the *Arabidopsis *gene, At-C4H (At2g30490), and a full length cDNA clone inserted into the pGreenII 62-SK vector (Fig. 1). As NADPH(+) HT-P450-reductase activity (EC 1.6.2.4) has been shown to be necessary for assaying HT-P450 activity in both bacteria [16] and yeast [9], we also identified a cDNA homologous to the *Arabidopsis *HT-P450 reductase (At4g30210), corresponding to Ad HT-P450 reductase, from kiwifruit and inserted it into pGreenII 62-SK.

### Transient expression of heme-thiolate P450

Initial experiments assessed the level of enzyme activity from transiently expressed Md-C4H1 and the effect of Ad-P450 reductase 10 days after *Agrobacterium *infiltration. The extent of conversion from cinnamic acid to p-coumaric acid, measured by HPLC, was similar for both the MdC4H1 and the MdC4H1 + Ad-P450 reductase, and significantly higher than for a transiently transformed empty vector control (data not shown). In addition, assaying *Agrobacterium *cultures just before infiltration did not reveal endogenous C4H enzyme activity in the bacteria. This suggested that the endogenous activity in the tobacco leaf provided sufficient NADP-dependent P450 reductase capacity and that additional transient expression of the reductase was not necessary. In this regard, transient assays in plants differ from other expression systems where an exogenous reductase is required for optimal enzymatic activity. The additional expression of the viral silencing suppressor P19 did not significantly increase the enzyme activity (data not shown). While expression of P19 has been shown to enhance the expression of other transiently expressed genes in other studies [5], in the case of HT-P450 it appears that gene expression levels are not impaired by gene silencing. This indicates that either the microsomal membrane surface area available to facilitate the reaction or the availability of the infiltration substrate is limiting

### Transient expression time course

Having established our ability to measure the conversion of cinnamic acid to p-coumaric acid, we further optimized the assay to determine the optimal period for substrate infiltration. The HPLC peak heights for both substrate (cinnamic acid) and product (p-coumaric acid) were plotted as a function of the time between substrate infiltration and extraction. The conversion of cinnamic acid to p-coumaric acid increased up to one hour, although subsequently the level of p-coumaric acid dropped (Fig. 2B). The concentration of cinnamic acid decreased over the whole time period tested. No p-coumaric acid was detected in the control. This decrease in the product may suggest that p-coumaric acid was modified by endogenous enzymes during that time. This is further supported by the appearance of a second product peak at an earlier retention time in the 2 h time point chromatograph.

We next investigated the time between infiltration of *Agrobacterium *and the substrate. Time points were chosen up to six days (with 60 min between substrate infiltration and extracting leaves) (Fig. 2C). At t = 0, there was also little conversion of cinnamic acid to p-coumaric acid, demonstrating a low endogenous C4H activity of the *N. benthamiana *leaf and the absence of C4H activity from the infiltrated *Agrobacterium *(Fig. 2C). The levels of un-reacted cinnamic acid declined as p-coumaric acid accumulated over the time period to six days (Fig. 2C). Thus it was concluded that six days' infiltration of *Agrobacterium *and 60 min infiltration of substrate provided a sensitive assay of HT-P450s.

We further verified the utility of the method with two other genes; *Actinidia deliciosa *galactose dehydrogenase, [17], and L-galactose-1-phosphate phosphatase [18]. Both genes expressed high levels of activity for their respective enzymes. In the case of galactose dehydrogenase, expression levels were ~100 times higher than P19 infiltrated control leaves after 7 days after infiltration, while the phosphatase showed high levels of expression of a L-Galactose-1-P specific phosphatase compared with undetectable activity in the control (data not shown).

### A vector for the analysis of promoter transcription factor interactions

**Figure 3:**
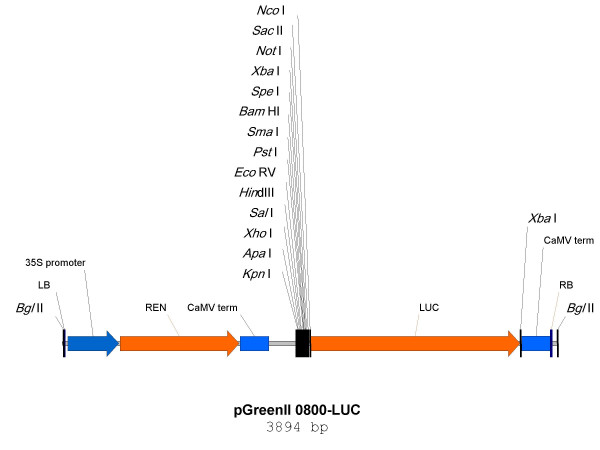
T-DNA region of the transient expression vector pGreenII 0800-LUC.

The promoter sequences for four chalcone synthase (CHS) genes (EC 2.3.1.74) from each of four species: *Arabidopsis*, apple (*Malus domestica*), pea (*Pisum sativum*) and petunia (*Petunia hybrida*) were each inserted into the multiple cloning site of pGreenII 0800-LUC (Fig. 3). In all cases the promoter was modified to introduce an *Nco*I site at the 3' end of the sequence (at the CHS initiation codon, ATG), allowing the promoter to be cloned as a transcriptional fusion with the LUC gene. Thus, TFs that bind the promoter and increase transcription could be identified by an increase in LUC activity. The promoters for *Malus domestica *MdCHS1 [GenBank: DQ026297] identified as a 1.3 Kb PCR fragment in the *Ecl*136II-linker-ligation library [genebank DQ022678], *Arabidopsis *CHS (TT4; AT5G13930 insert reference), petunia CHS-A [GenBank: X14591] and *Pisum sativum *CHS-1a [GenBank: X80007] were isolated from genomic DNA. In the same construct, a REN gene under the control of a 35S promoter provided an estimate of the extent of transient expression (Fig. 3). Activity is expressed as a ratio of LUC to REN activity.

The reporter construct, transformed into *Agrobacterium*, was mixed with an *Agrobacterium *strain carrying a 35S-MYB construct and co-infiltrated into tobacco leaves.

### Transient analysis of Transcription Factors

**Figure 4:**
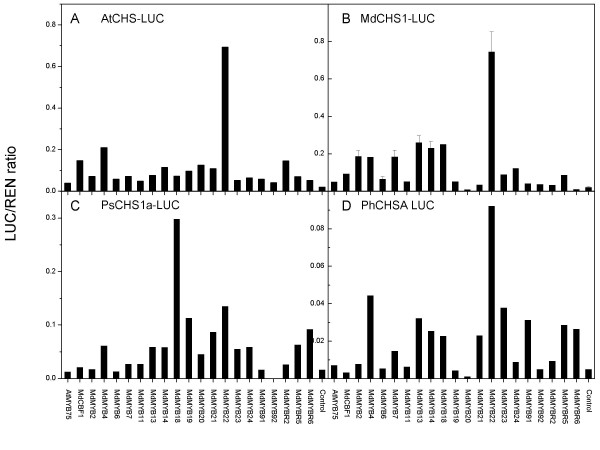
Relative LUC activity from transient expression analysis of 4 CHS promoters co-infiltrated with a plasmid containing genes for apple transcription factors fused to the 35S promoter. A, *Arabidopsis *CHS promoter (At5g13930); B, *Malus x domestica *CHS1 promoter (DQ022678); C, *Pissum sativium *CHS-1a promoter (X80007) and D, *Petunia hybrida *CHS-A promoter (X14591). Transcription factors: MdMYB2 [GenBank: DQ074459], MdMYB4 [GenBank: DQ074460], MdMYB6 [GenBank: DQ074461], MdMYB7 [GenBank: DQ074462], MdMYB11 [GenBank: DQ074463], MdMYB13 (GenBank: DQ074464), MdMYB14 (GenBank: DQ074465), MdMYB18 [GenBank: DQ074466], MdMYB19 [GenBank: DQ074467], MdMYB20 [GenBank: DQ074468], MdMYB21 [GenBank: DQ074469], MdMYB22 [GenBank: DQ074470], MdMYB23 (GenBank: DQ074471), MdMYB24 (GenBank: DQ074472), MdMYB91 [GenBank: DQ074473], MdMYB92 [GenBank: DQ074474], MdMYBR2 [GenBank: DQ074475], MdMYBR5 [GenBank: DQ074476], MdMYBR6 [GenBank: DQ074477], MdCBF1 [GenBank: DQ074478] and AtMYB75 (cDNA of At1g56650).

In the absence of a MYB TF, the LUC to REN ratio was low. This background level of activity presumably represents basal levels of MYBs present in tobacco leaves. In virtually all cases, the addition of a TF to the infiltration mixture increased the relative level of LUC activity compared with the background promoter activity in the absence of *Agrobacterium *containing a candidate TF (Fig. 4). The majority of TFs tested showed a similar, slightly higher than background, level of activity. We speculate that many of these TFs will have a low affinity to these promoter sequences, and thus cause a small non-specific *trans*-activation. We hypothesize that where the interaction between TF and promoter was more specific *trans*-activation, there is a significant increase in the LUC activity relative to REN (Fig. 4). For six of the promoter-TF comparisons, multiple repeats of the assay using six independently infiltrated leaves with the same *Agrobacterium *culture were used to generate the standard error of the data (Fig. 4B). Preliminary assays showed there were little day-to-day, plant to plant or leaf positional effects on the calculated ratio (data not shown). In addition, the concentration of *Agrobacterium *in the infiltration buffer did not affect the ratio significantly (data not shown). In these experiments an error of 14–17% was observed, similar to previous transient assay error estimations. We chose not to estimate the error for the remaining assay but set a threshold such that the REN value had to be above 100 relative light units (RLU) to be assured of a comparable degree of error in the remainder of the data.

The utility of the luciferase vector was demonstrated by identification of specific TFs that showed significantly higher LUC to REN ratios than background, compared with the average of other TFs. Instances where a promoter-TF interaction was identified usually resulted in at least ten times higher LUC to REN ratio than the average background compared with other MYBs, which showed a 1 to 2 fold increase in the relative LUC activity of the promoter alone. Thus we have identified from a pool of 20 transcription factors, those TFs likely to be involved in the regulation of the CHS gene (Fig. 4).

The ability of these transient assays to discriminate between strong and weak trans-activation was significantly influenced by the period of transient expression and the ratio of *Agrobacterium *carrying the transcription and luciferase reporter cassettes. Transient expression demonstrated clear distinctions between TFs after three to four days. However, prolonged transient expression over eight days (data not shown) reduced differences between TFs, suggesting an accumulation of TF protein to levels that are able to interact with a range of promoter sequences in a generic fashion, rather than with those target promoters that would be utilized *in vivo*. This promiscuous behavior is not surprising, given the sequence conservation within the R2R3 MYB DNA binding domain [19]. It is therefore important to note that the non-specific *trans*-activation in transient assays can be avoided by optimising the period of time between inoculation and assay.

We also found that increasing the ratio of the TF-containing *Agrobacterium *to the promoter-LUC-REN fusion containing *Agrobacterium *gave a clearer difference between high and low strength trans-activation. We were unable to determine TF interactions when the promoter-LUC fusion compromised less than 10% of the total infiltrate; this was because the correspondingly low luciferase activity made ratio measurements more variable.

There were significant *trans*-activation similarities between most of these CHS promoters; notably MdMYB22 [GenBank: DQ074470] was the strongest TF in *trans*-activating LUC in three of the CHS promoters tested: *Arabidopsis *(Fig. 4A), Petunia (Fig. 4D) and Apple (Fig. 4B). MdMYB22 has sequence homology to the maize P gene [GenBank: M73029] and AtMYB12 [AT2G47460] which has been shown to regulate genes involved in the flavanol biosynthesis pathway [20]. In addition MdMYB18 [GenBank: DQ074466] up-regulated LUC when fused to the Pea CHS-1a promoter (Fig. 4C).

**Figure 5:**
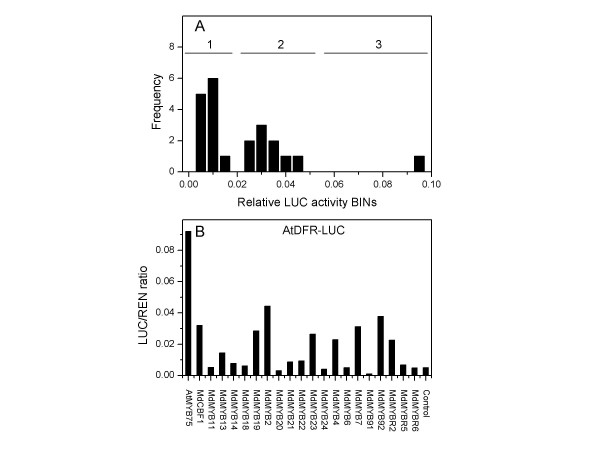
A, relative LUC activity from transient expression analysis of *Arabidopsis *DFR promoter (At5g42800), co-infiltrated with transcription factors as in Fig. 4. B, Frequency distribution of the relative LUC activity from Fig. 5A. Region 1; transient expression considered background promoter activity, region 2, partial promoter activations and region 3, strong promoter activation.

For each promoter tested there was a distribution of background *trans*-activation, which could be distinguished from a specific promoter interaction by the distribution of *trans*-activation (Fig. 5A). By examining the petunia CHS-A promoter data there appear to be three groups of TFs. Group 1 showed little or no *trans*-activation; this we take to be the background promoter activity in these assay conditions. Group 2 showed a normal distribution of activities significantly above background. This group included MdMYB18 [GenBank: DQ074466] and represents interactions we take to be poor or non-specific *trans*-activation. Group 3, in this case MdMYB22 [GenBank: DQ074470], was a TF that appears to have a specific *trans*-activation of the promoter. None of the apple MYB TFs was able to *trans*-activate the *Arabidopsis *dihydro flavonoid reductase promoter (TT3, At5g42800) (Fig. 5B), although AtMYB-75 (AtPAP1, At1g56650), which is known to regulate the anthocyanin pathway [21] was able to *trans*-activate this promoter fusion; this further demonstrates that the *trans*-activation profile for these CHS promoters was specific.

### Vector to investigate RNA silencing

**Figure 6:**
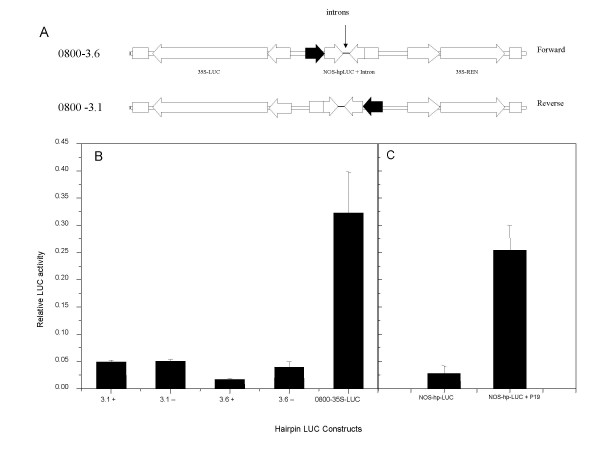
Transient expression analysis of hairpin constructs. A, pGreenII 0800-3.6 and pGreenII 0800-3.1 constructs contain the LUC and REN genes fused to the CaMV 35S promoter. Between these two reporter genes a LUC hairpin construct is fused to the NOS promoter in forward and reverse orientations. Both hp constructs contain an intron between the inverted repeats in both + and - orientation. B, Relative LUC activity for the pGreenII 0800-3.1 and 3.6 cassettes (for both +ve and -ve intron orientations) and pGreenII 1598-1, a LUC and REN construct with no hpLUC. C, The effect of P19 on silencing of the NOS-hpLUC cassette. The control (un-silenced) for this experiment is the 0800-35S-LUC in Fig 6B.

We have adapted the transient assay system in order to measure the effects on RNA silencing of the structure of the dsRNA hairpin and the influence of silencing suppressers. We were particularly interested in determining the role that RNA processing might play in efficient dsRNA production, and whether the presence of introns within hairpin cassettes can enhance the efficiency of RNA silencing activation [22]. Our RNA silencing assay system consists of the LUC and REN luciferase reporter genes in opposite orientation on a single T-DNA, each under the transcription regulation of the 35S promoter (Fig. 6A). Between the two reporter genes is a LUC hairpin sequence (hpLUC), under the control of the nopaline synthase (NOS) promoter. In the absence of a NOS-hpLUC hairpin both the LUC and REN activities accumulated (Fig. 6B).

To test the efficiency of transient silencing and to quantify the effect that an intron has on this efficiency, we built two constructs: pGreenII 0800-3.6 (+) and pGreenII 0800-3.6 (-), each including an intron in either splicable (+) or inverse (-) orientation of the hairpin (Fig. 6B). Both the NOS-hpLUC cassettes were effective in significantly reducing the level of LUC activity (Fig. 6B). This reduction in LUC activity was the result of the activation of RNA silencing because the co-infiltration of *Agrobacterium *expressing P19, a viral suppressor of gene silencing [5], was effective in overcoming the silencing effect of the LUC hairpin, and restoring LUC levels to those of the control (Fig. 6C).

Notably, the relative level of LUC in the hairpin construct that contained a functional, splicable intron was lower (0.0171 ± 0.0014) than from the equivalent cassette where the intron was in an inverted orientation (0.0394 ± 0.0099) such that it cannot be processed. This observation appears to be consistent with previous reports [22] which showed an increased in the percentage of transgenic plants with a gene silencing phenotype when the dsRNA hairpin constructs contained an intron. However, in these transient assays the absolute levels of LUC were very similar for both hairpin constructs, and the difference in relative luciferase level could also be achieved by elevating the levels of REN in the construct that contained the splicable intron. As the NOS-hpLUC cassette and the 35S-REN cassettes are in tandem, it is possible that the NOS promoter or the processing of the intron is influencing the transcriptional activity of the REN gene. To test this possibility we built and assayed a further two constructs, pGreenII 0800-3.1 (+) and (-) (Fig. 6B). In these constructs the NOS-hpLUC cassette directs transcription in the opposite orientation to that of pGreenII 0800-3.6, such that any cis-activation from the NOS cassette should influence 35S-LUC activity, but not 35S-REN activity. In transient assays using these constructs there was no significant difference between the hairpin with a splicable intron (0.0492 ± 0.0032) and the equivalent construct with an inverted intron (0.0504 ± 0.0029). This suggests that including an intron in the construction of a cassette that generates a dsRNA hairpin may function to influence the expression of any downstream tandem genes. In the case of the pHannibal vector, [13] this would correspond to the kanamycin resistance gene NptII, used for transformation selection. This suggests that the enhanced percentage of silencing described by Smith et al [22] may in part be due to enhanced recovery or stability of transgenic lines through improved expression of the kanamycin selection gene. We were unable to identify by RT-PCR, any RNA species that represented a hybrid RNA between the NOS promoter and either the LUC or REN gene, suggesting that the processing event may act as a transcriptional enhancer rather than effect the initiation of transcription at a distance.

### Measuring viral suppression of RNA silencing in

#### transient assays

**Figure 7:**
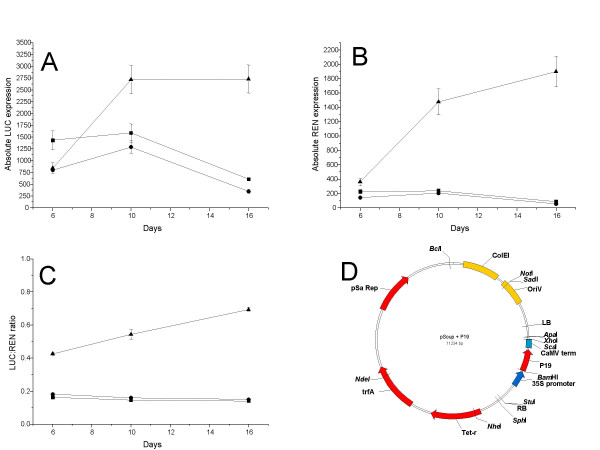
Absolute and relative LUC and REN expression levels from the pGreenII 1598-1 construct following 5,10 and 16 days infiltration into *N. Benthamina *leaves. (▲) pSoup helper plasmid, (■) pSoup0000 helper plasmid and (○) pSoup-P19 helper plasmid. A, Absolute LUC activity. B, Absolute REN activity. C, LUC to REN ratio. D, plasmid map of the pSoup-P19 helper plasmid.

After 4–5 days of *Agrobacterium *infiltration the levels of transient expression would be expected to drop because of the activation of RNA silencing resulting from the high level of gene expression [5]. The viral suppressor of RNA silencing, P19 from tomato bushy stunt virus (TBSV), is known to remove the small interfering (si)RNAs responsible for amplifying RNA silencing [23]. Transient expression can be enhanced and extended for several weeks by co-expressing P19 protein in the agro-infiltration mixture [5]. This is usually achieved by mixing different recombinant strains of *Agrobacterium *containing either the P19 construct or the gene of interest. The pGreen system requires an additional helper plasmid, pSoup, to enable binary plasmid replication in the *Agrobacterium *cell [24]. We introduced a P19 expression T-DNA into pSoup (Fig. 7), such that enhanced transient expression could be achieved without the need to mix *Agrobacterum *strains.

A time course of LUC and REN transient expression levels and the effect of including P19 in these assays is shown in Fig. 7. In all cases the reporter gene was pGreenII 0800 35S-LUC. Normally the transient assay data is expressed as a ratio of LUC/REN, reducing variability caused by leaf age, infiltrate volume and incubation conditions, variables known to strongly influence absolute reporter gene activity. However, in this experiment the ratio of LUC/REN luciferase expression masked the effect of P19 in transient assays, so the raw data from six infiltrated leaves are presented. Prior to day six, no difference was observed in assays with or without P19. Two-control plasmids were also tested; pSoup and pSoup0000. pSoup does not contain a T-DNA while pSoup0000 includes a minimal T-DNA region that shares some sequence homology with the pSoup-P19 T-DNA. Both the pSoup and pSoup0000 helper plasmids showed a similar and predicted loss of reporter gene activity from day six. The loss of absolute activity was most noticeable in the LUC assay, probably as a result of the instability of the LUC protein relative to the REN protein. There was no difference in the expression profile from these two control plasmids, demonstrating a lack of interaction between the T-DNA in pSoup0000 and the luciferase reporter cassette T-DNA in pGreenII 0800-35S-LUC. We can therefore conclude that the differences seen in the pSoup-P19 analysis are due to the presence of the P19. From day 6, and most noticeably by day 14, the absolute levels of both LUC and REN were significantly higher in the presence of pSoup-P19. This suggests that for vectors constructed using pGreenII, the use of pSoup-P19 is the most convenient means for ensuring high levels of transient gene expression where significant levels of protein are requited.

## Conclusion

We have presented a simple method using transient expression of genes in tobacco to test for a range of gene functions. Vectors have also been developed to exclude the transformation selection genes present in many binary vectors. Removing the transformation selection reduced plasmid size and improved cloning efficiency. We described a method to characterize the enzymatic activity of proteins encoded by genes cloned into these *Agrobacterium *vectors, a method to screen the relative transcriptional activities of TFs, and a method to study gene silencing through dsRNA. By incorporating both luciferase and renilla chemiluminescent reporter genes on a single T-DNA we are able to effectively reduce the intrinsic variability of leaf infiltration, allowing reproducible promoter activity determinations.

## Materials and methods

*Nicotiana benthamiana *plants were grown in a glasshouse at 22°C and using natural light with daylight extension to 16 h. Plants were grown until they had six leaves and the youngest leaves over 1 cm long were infiltrated with *Agrobacterium *and maintained in the glasshouse for the duration of the experiment.

*Agrobacterium tumefaciens*, GV3101(MP90) [25] were cultured on Lennox agar (Invitrogen) supplemented with 50 μg.ml^-1 ^kanamycin (Sigma) and incubated at 28°C. A 10 μl loop of confluent bacterium were re-suspended in 10 ml of infiltration media (10 mM MgCl_2_, 0.5 μM acetosyringone), to an OD_600 _of 0.2, and incubated at room temperature without shaking for 2 h before infiltration. Infiltrations were performed according to the methods of Voinnet *et al*. (2003) [5]. Approximately 300 μl of this *Agrobacterium *mixture was infiltrated into a young leaf of *N. benthamiana *and transient expression was assayed from three to 14 days after inoculation.

Each of the promoter-LUC fusions in pGreenII 0800-LUC were used in transient transformation by mixing 100 μl of *Agrobacterium *transformed with the reporter cassette and 900 μL of a second *Agrobacterium *strain transformed with a cassette that contained the TF gene fused to the 35S promoter in either a pART27-derived or pGreenII 62-SK binary vectors described below.

### Oligonucleotides

RPH-138: GTGAGAGGTCCTAAGCTTATGTCCGGTTAT; RPH-139: CTTGACTGGCGAGAATTCCCACGATCTCTTT; RPH-140: ATTGACAAGGATGGATCCCTACATTCTGGA; RPH-141: CACGATCTCTTTTTCCGTCATCGTCT; RPH-146: TGGCCTTTATGAGGGAATTCCTGATTTTTC; RPH-179: TGGCGGTTTTGGTACCCCGGGTCAAC; RPH-180: CCATCACCATGGTAGTATACACCAAC; RPH-198: CACACAGTTGGGAGGAGTTGCTGTCCC; RPH-199: CTTGCGAACTTCTTCGACGGTCACCAT; RPH-212: AATTGGTACCGATATCGAGCTC; RPH-213: AGCTGAGCTCGATATCGGTACC; RPH-332: ACTCCTCGACTGTCACCATGGTTGCTTG; RPH-333: GCGGAAGGGTACCGAATTCATAGCAACTGG

### Construction of pGreenII-62-SK

The cauliflower mosaic virus (CaMV) 35S expression cassette (*Kpn*I-*Bgl*II) of pJIT-62 [26] was inserted into the multiple cloning site (MCS) (*Kpn*I-*Bam*HI) of pGreenII-0000 [24]. Flanking sequences from the LB to the 35S-promoter and from the CaMV-terminator to the RB were deleted by digestion, (*Stu*I-*Kpn*I and *Spe*I-*Hpa*I respectively), T4 pol and re-ligation to produce pGreenII-62-1. The pUC9 MCS was converted to the pBluescript MCS by ligating two oligonucleotides (RPH-212 and RPH-213) to the *Eco*RI-*Hind*III cut vector, generating *Kpn*I and *Sac*I sites, then inserting a *Kpn*I-*Sac*I fragment from pBluescript SKII+ (Stratagene).

### Construction of the plasmids containing genes for enzymes and TFs

The HortResearch apple EST libraries were constructed in either the Lambda ZAP Express or the Lambda ZAP II vector systems (Stratagene), resulting in cDNAs cloned unidirectionally as *Eco*RI-*Xho*I fragments in pBK-CMV or pBluescript SK^-^, respectively.

Genes encoding various enzymes and transcription factors were cloned into one of two plant expression vectors derived from the pART7/27 binary vector system [27]. Both vectors carry the same transcriptional regulatory signals for plant gene expression, namely the CaMV35S promoter and octopine synthase terminator. The T-DNA border elements, chimeric kanamycin selectable marker and vector backbone are identical in both vectors. The two derivatives differ in that one is suitable for conventional restriction enzyme cloning of cDNAs whilst the second facilitates Gateway recombination cloning of the cDNAs.

In order to improve the compatibility of restriction sites in the multiple cloning site of pART7 with those of the pBK-CMV or pBluescript SK^- ^EST library vectors, the *Xho*I-*Xba*I region of pART7 was replaced with the *Sal*I-*Xba*I multiple cloning site region of pBK-CMV, generating pSAK7. cDNAs from the EST libraries were cloned into pSAK7 as either *Eco*R1-*Xho*I or *Bam*HI-*Xho*I fragments, placing them under the transcriptional control of the CaMV 35S promoter. The 35S-cDNA-*ocs*3' cassette was then cloned as a *Not*1 fragment into pART27, to generate the plant gene expression construct.

Where conventional cloning was more problematic, due to the lack of suitable restriction sites, a Gateway-adapted version of the pART7/27 plant transformation system was utilised. This Gateway-adapted version was produced by cloning the CaMV35S promoter, multiple cloning site and octopine synthase transcriptional terminator cassette of pART7 as a *Not*I fragment into pART27. Subsequently, the 1711 bp Gateway RfA cassette (Invitrogen Corp.) was cloned into the *Sma*I site of the multiple cloning site to generate pHEX2 (35S-*att*R1-Cm^R^-*ccd*B-*att*R2-*ocs*3'). cDNAs from the EST libraries were cloned into pHEX2 using Gateway recombination technology and all Gateway reactions were performed as recommended by the manufacturer (Invitrogen Corp.). cDNAs were amplified using universal primers designed to the multiple cloning site regions of the pBK-CMV or pBluescript SK^-^. The primers used for pBluescript SK- clones were 5-GGGGACAAGTTTGTACAAAAAAGCAGGCTCCCCGGGCTGCAGGAATTC-3' and 5'- GGGGACCACTTTGTACAAGAAAGCTGGGTCCGGGCCCCCCCTCGAG-3' and the primers used for pBK-CMV clones were 5'-GGGGACAAGTTTGTACAAAAAAGCAGGCTTCGACACTAGTGGATCCAAAGAATTC-3' and 5'-GGGGACCACTTTGTACAAGAAAGCTGGGTGCCGCTCTAGAAGTACTCTCGAG-3'

Amplification with these primers results in PCR products with *att*B ends which were recombined with the *att*P sites of the Gateway pDONR201 vector, creating pENTRY vectors. All pENTRY vector clones were sequence verified to ensure the fidelity of the cDNA sequence. Gateway *att*P × *att*R reactions were then carried out with the pENTRY vector and the pHEX2 Destination vector, to generate the plant gene expression construct.

Enzyme expression plasmids. The apple cDNA clone of MdC4H1 [GenBank: DQ075002] was inserted into pGreenII 62-SK as an *Eco*RI-*Xho*I fragment. The genes for L-galactose dehydrogenase [GenBank: AY176585] and L-galactose-1-P phosphatase [GenBank: AY787585] from kiwifruit were cloned as previously described [18] and transformed into *Agrobacterium *using standard methods.

### Construction of pGreenII 0800-LUC

The *Renilla *reporter gene pRL-null (Promega, Madison, WI) was modified to remove the mammalian 5'UTR intron by digestion with *Nhe*I-*Spe*I, T4 Pol, followed by re-ligation. This gene was inserted into the expression cassette p35S-2 [24], and the flanking sequences deleted as above. This cassette was inserted into the *Hpa*I site of pGreenII 0000, to produce pGreen 0800-1. A 35S-LUC-expression cassette was inserted into pGreenII 0800 to produce pGreenII 0800-35S-LUC. The flanking sequence between the CaMV terminator and the RB was deleted (*Spe*I-*Stu*I, T4 pol, ligation). Finally the pBluescript MCS replaced the 35S promoter to produce pGreenII 0800-LUC. These vectors are available on request.

### Promoter cloning into pGreenII 0800-LUC

A 0.97 kb region of the *Arabidopsis *CHS promoter (At5g13930) was amplified by PCR from the *Arabidopsis *ecotype Columbia using the primers RPH-179 and RPH-180, then digested with *Kpn*I and *Nco*I and cloned into the MCS of pGreenII 0800-LUC. The 1.04 kb pea CHS-1a promoter [GenBank: X80007] was subcloned into pGreenII 0800-LUC as an *Eco*RI-*Nco*I fragment [28]. A 0.92 kb Petunia CHS-A promoter [GenBank: X14591] was amplified by PCR using primers RPH-332 and RPH-333 from a V26 genomic DNA, digested with *Kpn*I and *Nco*I and cloned into pGreenII 0800-LUC. The 1.3 kb Apple CHS1 promoter [GenBank: DQ022678] was isolated from *Malus domestica *Royal Gala, using the Genome Walker kit (Clonetech) with gene specific primers RPH-198 and RPH-199, then cloned into pGEM T-easy (Promega) and subcloned as a *Sal*I-*Nco*I fragment into pGreenII 0800-LUC. *pwo*-Polymerase (Roche) was used for all PCR amplifications and cloned genes were sequenced to confirm no sequence modifications were incorporated.

### Construction of hp-cassette

A short hairpin was built by asymmetric amplification of a region of the LUC gene: primers RPH-138 and RPH-146 were used to amplify a 454 bp fragment introducing a *Hin*dIII and *Eco*RI site near the end of the PCR product. The second PCR product corresponding to the antisense region of the hairpin was a 283 bp PCR product of amplification using primers RPH-140 and RPH-139. The first primer, RPH-140, was from the same region as RPH-138 but introduced a *Bam*HI site near the end of the PCR product. RPH-139 was 142 bp closer to RPH-138 and RPH-140 than RPH146, and also introduced an *Eco*RI site near the end of the amplified product. The PCR products were digested with appropriate restriction enzymes and these two digested PCR products were used in a 3-way ligation to *Hin*dIII-*Bam*HI cut pNOS-7 [24] to produce the NOS-hpLUC cassette. This NOS-hpLUC was modified to replace the loop region with an intron by amplifying the NOS-hpLUC cassette with primer RPH-141, common to both the sense and antisense region, with *pwo*-polymerase. An intron sequence from a kiwifruit terpene synthase genes [GenBank: DQ026298] was also amplified with primers RPH-099 and RPH-100. The intron amplification product was treated with polynucleotide kinase (NEB) and used in a ligation with the non-phosphorylated amplification product of the NOS-hpLUC cassette. The resulting cassettes either contained an intron in +ve (RPH-099 to RPH-100 relative to the NOS promoter) or -ve (RPH-100 to RPH-099 relative to NOS promoter) configuration. Accordingly the +ve intron configuration had a splicable GT to AG intron arrangement, the -ve intron configuration did not. The nos-hpLUC cassette and intron containing derivatives were inserted into a 35S-LUC, 35S-REN cassette; pGreenII 0800 35S-LUC, as an *Eco*RV fragment. In this way the reporter cassettes were generated with the intron containing NOS-hpLUC cassette in both orientations. pGreenII 0800-3.6 had the NOS promoter directing transcription in the same orientation as the 35S-REN and towards the T-DNA Right border (forward). pGreenII 0800-3.1 had the NOS promoter directing transcription of the hpLUC in the same orientation as the 35S-LUC and towards the T-DNA left border (reverse) (Fig. 6A).

### Construction of pSoup-P19

The pSoup helper plasmid [24] was converted to a T-DNA carrying version by inserting a *Bgl*II fragment that contained the T-DNA from pGreen0000 into the unique *Bam*HI site of pSoup creating pSoup0000. A 35S-P19-CaMV fusion was isolated from pBIN-61-P19 [5] as a *Sac*I-*Bgl*II partial and inserted into the pSoup0000 multiple cloning site to produce pSoup-P19.

### Enzyme assays

#### C4H assay

0.5 mM cinnamic acid, pH 7.0 (*trans*-cinnamic acid 99+%, Aldrich, Milwaukee) was infiltrated into the tobacco leaves after *Agrobacterium *infiltration and allowed to incubate in the leaf for up to 2 hours before extraction.

The infiltrated leaves were removed from the *N. benthamiana *plant and frozen in liquid nitrogen. The material was crushed in a pestle and mortar before extraction with 10 ml of ethyl acetate for 18 hours. Post extraction, the plant material was removed by filtering and the organic solvent evaporated. The residue was re-dissolved in 1 mL methanol and used for HPLC analysis.

Twenty μl of the methanolic extracts were run on a HP1100 HPLC system on a Vydac^® ^RP 300Å C18 HPLC column (250 × 4.6) (Phenomenex, CA, USA). The mobile phases were A: 1% phosphoric acid and B: 100% acetonitrile (BDH). The separation of the product and the substrate was performed using gradient elution [29] at room temperature with a flow rate of 1 ml/min. The substrate and product were monitored using UV at 275 and 310 nm. The peak areas were quantified with cinnamic and p-coumaric acid (*trans *98%, Aldrich, Milwaukee) standards.

#### Galactose dehydrogenase and Galactose-1-P phosphatase assays

Tobacco leaves were harvested approximately 70 hours after infiltration with *Agrobacterium *containing the plasmid encoding the respective genes under control of the 35S promoter. Tissue was frozen in liquid nitrogen, protein was extracted as described by [30] and assayed as described by [18,17].

**Firefly Luciferase and *Renillia *luciferase **were assayed using the dual luciferase assay reagents (Promega, Madison, USA). After inoculation and a transient incubation of 2–4 days, 2 cm leaf discs were harvested and ground in 500 μl of Passive Lysis Buffer. Five μl of a 1/100 dilution of this crude extract was assayed in 40 μl of Luciferase Assay Buffer, and the chemiluminescence measured. 40 μl of Stop and Glow™ buffer was then added and a second chemiluminescence measurement made. Absolute RLU were measured in a Turner 20/20 luminometer, with a 5 second delay and 15-second measurement. Data was collected as ratio or, for multiple data points (e.g. several leaves of different ages were infiltrated), the regression-gradient and regression-standard-error were used as a measure of relative promoter strength. Ratios are without units as both the light measurement and protein concentrations are identical. Background controls were run with only the promoter-LUC, 35S-REN reporter plasmid (no TF). In some cases, positive controls were run using a TF with known activity.

## Authors' contributions

RPH for experimental design, vector construction, promoter Isolation, data collection and manuscript preparation.

ACA and WAL for transcription factor mining, data analysis and manuscript preparation.

ENF and MDT for mining, enzyme analysis, data collection and manuscript preparation.

APG for vector strategy design, sequence editing and assembly, manuscript preparation

SK, KG and KB for vector construction and microbiology.
